# CeO_2_-Supported Pt Catalysts Derived from MOFs by Two Pyrolysis Strategies to Improve the Oxygen Activation Ability

**DOI:** 10.3390/nano10050983

**Published:** 2020-05-21

**Authors:** Xueqing Zhu, Hui He, Yanxia Li, Haoyuan Wu, Mingli Fu, Daiqi Ye, Junliang Wu, Haomin Huang, Yun Hu, Xiaojun Niu

**Affiliations:** 1 School of Environment and Energy, South China University of Technology, Guangzhou 510006, China; 201720141893@mail.scut.edu.cn (X.Z.); eshehui@mail.scut.edu.cn (H.H.); 201821035341@mail.scut.edu.cn (Y.L.); 201630793250@mail.scut.edu.cn (H.W.); cedqye@scut.edu.cn (D.Y.); ppjl@scut.edu.cn (J.W.); huanghm@scut.edu.cn (H.H.); huyun@scut.edu.cn (Y.H.); xjniu@scut.edu.cn (X.N.); 2 Guangdong Provincial Key Laboratory of Atmospheric Environment and Pollution Control, Guangzhou 510006, China; 3 National Engineering Laboratory for VOCs Pollution Control Technology and Equipment, South China University of Technology, Guangzhou 510006, China

**Keywords:** metal organic frameworks, functional derivatives, pyrolysis strategies, toluene oxidation, oxygen activation abilities

## Abstract

Functional metal organic framework (MOF) derivatives have attracted tremendous attention as promising catalysts for various reactions. The thermal decomposition strategies have a vital effect on the structures and physicochemical properties of functional MOF derivatives. Nevertheless, what effect does the pyrolysis strategy have on MOF derivatives need further study. In this work, one-step (under dry air) and two-step (first under N_2_ and then dry air) pyrolysis are chosen to prepare the functional ceria-based MOF derivatives with novel hierarchical pore structure. In comparison with the derivatives prepared by one-step pyrolysis, the two-step pyrolysis composites exhibit better catalytic activity for toluene oxidation due to the higher contents of surface absorbed oxygen species and surface oxygen vacancies. The reusability and durability test demonstrates perfect stability of such functional MOF derivatives. The in-situ UV Raman reveals that two-step strategy is favorable for enhancing the gaseous oxygen activation ability of the functional MOF derivatives. Those findings may instruct the synthesis of functional MOF derivatives via different pyrolysis strategies as well as afford a further understanding of the crucial role of oxygen vacancies.

## 1. Introduction

Toluene, a typical aromatic volatile organic compound (VOC), is considered as an important kind of carcinogen, which can pose a health hazard to human beings. Thus, the elimination of toluene is a worldwide research focus in the environmental and healthy safety field. Noble-metal nanoparticles (NPs), especially Pt NPs, have been attracting enormous research interest for decades due to their fascinating catalytic performance for toluene oxidation [1,2]. Unfortunately, because of high surface energies, these noble-metal NPs often suffer from serious aggregation and fusion during reactions, which results in significant loss of catalytic activities. Recently, a large number of research efforts have been fighting with the sintering effect, and using porous materials as carriers for NP immobilization which is regarded as a promising strategy to solve the problem of NP aggregation [3].

Metal organic frameworks (MOFs), a new class of porous crystalline materials constructed by metal nodes and organic linkers, have been widely used in gas storage [4], chemical separation [5], drug delivery [6], and heterogeneous catalysis [7]. In particular, benefitting from the high surface areas and uniform pore structures, MOFs have recently emerged as novel host matrices to offer a platform for immobilizing NPs within MOFs [8,9,10]. These NP@MOF composites exhibit excellent chemoselectivity owing to the molecular sieving effect from the frameworks as well as reduce NPs aggregation effectively, but the embarrassed situation is the poor reaction efficiency [11,12,13]. Currently, the research interests are gradually transfering from NP@MOF composites to functional MOF-based derivatives for improving the catalytic activity [14]. For example, Huo and co-workers have reported a simple strategy to design Pt@Co_3_O_4_ by encapsulation of Pt NPs in ZIF-67 and high-temperature calcination, which exhibited excellent catalytic activity and high stability for CO oxidation reaction [15]. Based on a great deal of research, the thermal decomposition strategy has a crucial effect on the properties of MOF derivatives [16]. Zheng and co-workers have obtained Co_3_O_4_ from ZIF-67 with diverse morphologies by different pyrolysis strategies [17]. The majority of recent research about pyrolysis strategy focuses on the change of surface morphology. The influence on physicochemical property, however, should be further studied. And the latter is an important factor in catalytic reaction. Therefore, it is necessary to develop new insights into the influence on physicochemical property of MOF derivatives via different pyrolysis routes.

Gaseous oxygen activation ability is one of the most essential physicochemical properties in gas–solid interface reaction, and recent studies highlight that surface oxygen vacancies are closely related to the activation of gaseous oxygen [18,19]. However, the essential roles of surface oxygen vacancies for gaseous oxygen activation are still ambiguous. Furthermore, the absence of quantitative research on gaseous oxygen activation ability restricts the further understanding of oxygen activation on catalysts surface. Thus, the detailed investigations of surface oxygen vacancies and gaseous oxygen activation are imperative. In situ UV Raman offers an opportunity to characterize catalysts in real time, which is helpful in comprehending the oxygen activation process and conducting the quantitative analysis.

Here, the functional ceria-based MOF derivatives with hierarchical pore structure are synthesized by two different kinds of pyrolysis strategies, one-step pyrolysis versus two-step pyrolysis, to realize effective immobilization of Pt NPs. The obtained functional MOF derivatives as heterogeneous catalysts exhibit outstanding catalytic activity and perfect stability for toluene oxidation. In situ UV Raman demonstrates that the gaseous oxygen activation ability of the functional MOF derivatives has been promoted by a two-step strategy, which plays an important role in enhancing toluene oxidation.

## 2. Materials and Method

### 2.1. Catalyst Preparation

Chemicals: All chemical materials used were obtained commercially and used without further purification. Cerium nitrate hexahydrate (Ce(NO_3_)_3_∙6H_2_O, 99.95% metals basis), trimesic acid (1,3,5-H_3_BTC, 98%), chloroplatinic acid hexahydrate (H_2_PtCl_6_∙6H_2_O, AR), polyvinylpyrrolidone (PVP, M_W_ = 58000), *N*,*N*-Dimethylformamide (DMF, 99.5%), ethanol (99.7%), ethylene glycol (98%), and n-hexane (97%) were supplied by Aladdin (Shanghai, China). Acetone (99.5%) was supplied by Guangzhou Chemical Reagent (Guangzhou, China). Ultrapure water (18.2 MΩ cm) was produced by a Millipore purification system.

Preparation of PVP-stabilized Pt NPs: PVP-stabilized Pt NPs were synthesized by an alcohol reduction that reported elsewhere [20,21]. Briefly, we used Pt(EG) to represent the Pt NPs reduced by ethylene glycol and Pt(Et) was reduced by ethanol. In a typical synthesis, Pt(EG) NPs were prepared by refluxing a mixture of PVP (222 mg), H_2_PtCl_6_∙6H_2_O (50.75 mg), and ethylene glycol (20 mL) in a 100 mL flask at 180 °C for 10 min. The as-synthesized Pt(EG) NPs in the mixed solution were precipitated by acetone and subsequently collected by centrifugation at 8000 rpm for 5 min. The sample was cleaned with acetone and n-hexane to remove excess free PVP, and dispersed in DMF with the concentration of 1 mg mL^−1^. Pt(Et) NPs were synthesized via refluxing of 50 mL ethanol solution containing 33.3 mg PVP and 15.54 mg H_2_PtCl_6_·6H_2_O in a 100 mL flask at 120 °C for 180 min. Then, excess ethanol was removed by rotary evaporator. Then, excess ethanol was removed by rotary evaporator. Furthermore, the following steps including precipitation, centrifugation, and purification were the same as that for Pt(EG) NPs.

Preparation of Ce-BTC and Pt@Ce-BTC: Ce-BTC was synthesized via a solvothermal method [22]. Typically, Ce(NO_3_)_3_∙6H_2_O (5 mmol) and H_3_BTC (10 mmol) were dissolved in a solvent mixture of DMF (50 mL) and ultrapure water (10 mL). Then the stock solution was transferred into a 100 mL Teflon-lined stainless steel autoclave and kept at 100 °C for 24 h. After the solvothermal reaction, the crystalline powder was collected by filtration and washed several times with DMF to remove unreacted reactants. Finally, pure Ce-BTC crystalline powder was collected after drying in a vacuum oven at 100 °C for 12 h. Specially, 5 mL of DMF containing 5 mg Pt NPs were added into the stock solution with stirring for 10 min, and the dark brown solution was transferred into 100 mL Teflon-lined stainless steel autoclave. The reaction condition was the same as that for Ce-BTC. Eventually, the brown crystalline powder was denoted as Pt(EG)@Ce-BTC or Pt(Et)@Ce-BTC, herein, Pt(EG) and Pt(Et) showed the same meaning as above.

Preparation of CeO_2_ and Pt@CeO_2_: CeO_2_ and Pt@CeO_2_ were prepared via one-step in situ pyrolysis of as-synthesized Ce-BTC or Pt@Ce-BTC. In a typical process, Ce-BTC or Pt@Ce-BTC was treated at 350 °C for 6 h under dry air with a flow rate of 100 mL min^−1^ in the tube furnace. After that, the obtained samples were named as CeO_2_ (Air), Pt(EG)@CeO_2_ (Air), and Pt(Et)@CeO_2_ (Air), respectively. Moreover, CeO_2_ and Pt@CeO_2_ were also prepared by a two-step pyrolysis route. The MOF precursors were firstly pyrolyzed at 500 °C for 5 h under N_2_ and for another 3 h under dry air at 300 °C. Herein, the two-step pyrolysis samples were named as CeO_2_ (N_2_-Air), Pt(EG)@CeO_2_ (N_2_-Air), and Pt(Et)@CeO_2_ (N_2_-Air), respectively. 

### 2.2. Activity Measurement

Catalytic activities of the samples were evaluated in a continuous flow micro-reactor made of quartz with 6 mm internal diameter. 200 mg catalysts (40–60 mesh) were diluted with 800 mg quartz sand. The mixed particles were packed at the bed of the reactor. Before the activity evaluation, the samples were reduced under 10% H_2_/N_2_ at 350 °C for 3 h. In reusability test, before each run of the activity evaluation, the samples were reduced under 10% H_2_/N_2_ at 350 °C for 3 h. The total flow rate of the reactant mixture (100 ppm Toluene, 20% O_2_/N_2_) was 80 mL min^−1^, corresponding to the weight hourly space velocity (WHSV) at 24,000 mL g^−1^ h^−1^. The concentrations of the reactants and products were analyzed by an on-line gas chromatograph (GC-2014C, Shimadzu, Japan) equipped with FID and the concentration of CO_2_ in the outlet gas was monitored by another FID with a conversion furnace to convert CO_2_ to CH_4_.

Toluene conversion (*X*, %) was calculated according to Equation (1)

*X* = (1 − *C_out_(Toluene)/C_in_(Toluene)*) × 100% (1)

where *C_in_(Toluene)* was the inlet concentration of toluene and *C_out_(Toluene)* was the outlet concentration.

Apparent activation energies were estimated using Arrhenius plots calculated by Equation (2) for toluene conversions lower than 10%

*Lnr* = −*E_a_/(RT) + lnA* (2)

where *r*, *E_a_* and *T* were reaction rate (mol g^−1^ s^−1^), apparent activation energy (kJ mol^−1^), temperature (K), respectively.

### 2.3. Catalyst Characterization

Thermogravimetric analysis (TGA) was performed on a STA 449 system (Netzsch, Bavaria, Germany) under N_2_/dry air flow with a heating rate of 5 °C min^−1^ from room temperature to 600 °C.

X-ray diffraction (XRD) patterns were recorded with a D8 ADVANCE diffractometer (Bruker, Billerica, MA, USA) using Cu Kα radiation source (*λ* = 1.5418 Å) at a scanning speed of 4° min^−1^ over the 2*θ* range of 5–50° for MOF precursors and 20–90° for pyrolysis samples.

Nitrogen adsorption–desorption isotherms were measured at 77 K on an ASAP 2020 instrument (Micromeritics, Norcross, GA, USA). Before the measurement, each sample was evacuated at 250 °C for 3 h.

Scanning electron microscopy (SEM) images were taken by a Quanta 650 (FEI, Hillsboro, OR, USA) at an accelerating voltage of 20 kV.

Transmission electron microscopy (TEM) images were obtained using a Tecnai G^2^ F20 S-TWIN microscope (FEI, Hillsboro, OR, USA) with an EDX detector operated at 200 kV.

The Pt loading in the MOF derivatives were quantified by ICP-MS on Agilent 7500ce (Agilent, Palo Alto, CA, USA).

X-ray photoelectron spectroscopy (XPS) analysis was performed with an Escalab 250Xi (ThermoFisher Scientific, Waltham, MA, USA) using Al Kα excitation source (*hv* = 1486.8 eV), and the binding energy values were calibrated using the C 1s peak (*hv* = 284.6 eV) as reference.

Room temperature UV Raman was obtained in a LabRAM HR Evolution Laser Raman Spectrometer (HORIBA, Paris, France) with a CCD detector and a He-Cd laser source (325 nm). The spectrum was recorded by two 180 s periods of subsequent laser exposure. In situ UV Raman spectra were achieved at every 10 °C from 80 °C to 170 °C with a ramp rate of 1 °C min^−1^ under N_2_ or dry air, respectively.

## 3. Results and Discussion

### 3.1. Structural Analysis

Ce-BTC (BTC = 1,3,5-benzenetricarboxylate) and Pt@Ce-BTC are prepared via a solvothermal method. The structure, porosity, and thermostability of the as-obtained MOFs have been studied. The X-ray diffraction (XRD) pattern of as-synthesized Ce-BTC matches well with the simulated pattern confirming good crystallinity and purity (Figure S1, Supporting Information) [23]. Compared with pristine Ce-BTC, no significant loss of crystallinity can be detected in the XRD patterns of Pt@Ce-BTC (Figure S1, Supporting Information), which implies that the immobilization of Pt NPs does not affect the integrity of Ce-BTC frameworks. Furthermore, the absence of Pt NP characteristic peaks could probably be related to the confinement effect by Ce-BTC frameworks. According to the nitrogen sorption measurements, all the samples exhibit classical type Ι isotherms with almost no hysteresis (Figure S2, Supporting Information), displaying the main characteristic of microporous materials. BET surface areas for Ce-BTC, Pt(EG)@Ce-BTC, and Pt(Et)@Ce-BTC are 762, 745, and 758 m^2^ g^−1^, respectively, which are comparable to the reported values [24]. The tiny decrease in surface area and total pore volume of Pt@Ce-BTC compared with intrinsic Ce-BTC is mainly due to the contribution of nonporous Pt NPs (Table S1, Supporting Information). Thermogravimetric analysis (TGA) curve shown in Figure S3 (Supporting Information) reflects that the Ce-BTC framework is thermally stable up to 350 °C. Obviously, it can be seen that two major weight losses are observed from ambient temperature to 600 °C. Typically, the first stage below 170 °C is attributed to the desolvation process while the second sharp weight loss above 350 °C corresponds to the collapse of Ce-BTC framework. In a word, pure microporous Ce-based MOFs are successfully synthesized by solvothermal method.

**Figure 1:**
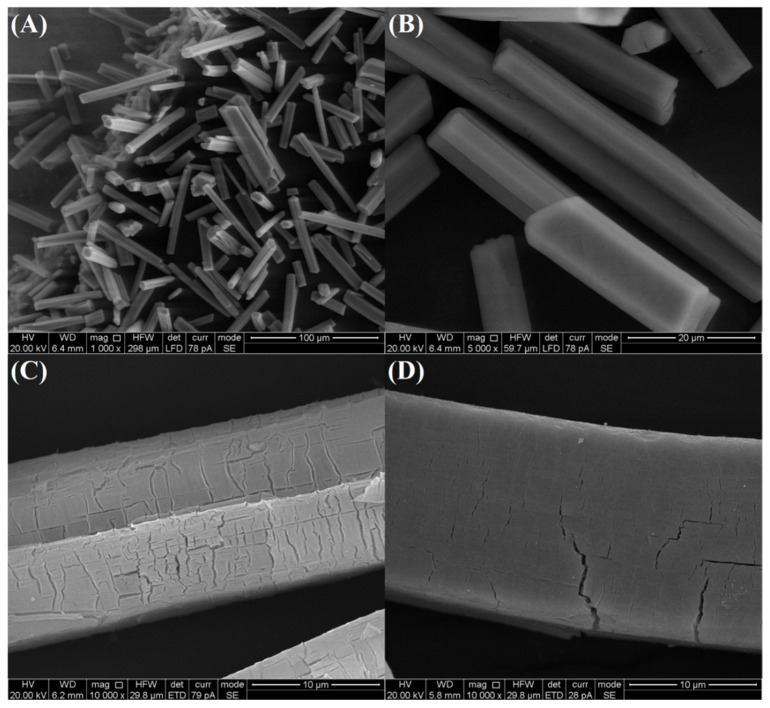
SEM images of (**A**,**B**) as-synthesized Ce-BTC, (**C**) CeO_2_ (Air) and (**D**) CeO_2_ (N_2_-Air).

As revealed by the scanning electron microscopy (SEM) images in Figure 1A,B, the Ce-BTC is microrod structure with smooth clean surfaces, and the sizes of 50–75 μm in length and 5–10 μm in diameter. After pyrolyzing, the original shape and size of as-synthesized Ce-BTC can be well-maintained in the MOF derivative products (Figure 1C,D). However, in sharp contrast with the smooth clean surface of the Ce-BTC crystals, the surfaces of CeO_2_ (Air) and CeO_2_ (N_2_-Air) are fairly rough. Cracks and fissures are generated during the pyrolysis process, which are attributed to the heterogeneous shrinkage phenomenon caused by the decomposition of organic ligands [17]. By comparing the SEM images of CeO_2_ (Air) and CeO_2_ (N_2_-Air), it can be seen that CeO_2_ (N_2_-Air) possesses fewer fissures than CeO_2_ (Air), due to the mild oxidation decomposition by two-step pyrolysis route.

**Figure 2:**
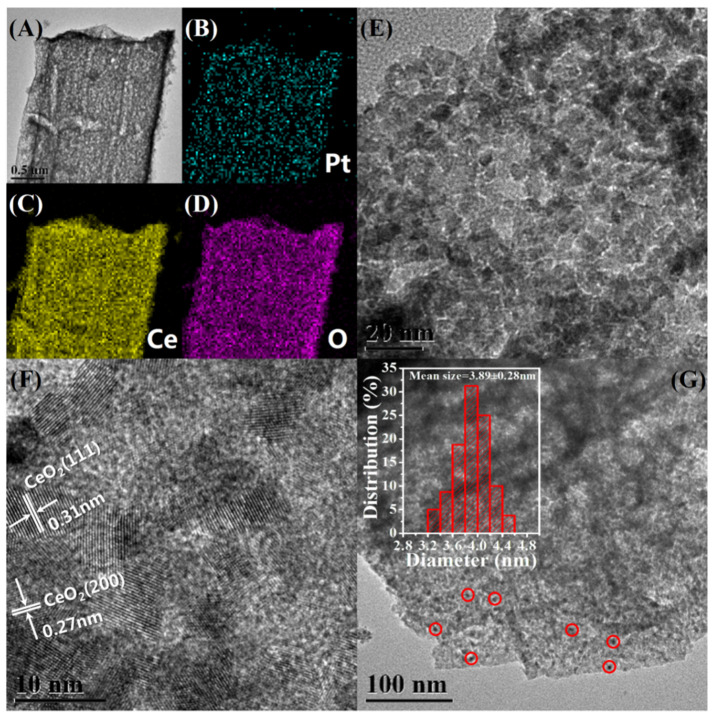
Morphology and structure of Pt(EG)@CeO_2_ (Air). (**A**,**E**) TEM images, (**F**) HRTEM images, (**G**) the distribution of Pt NPs and the inset is histogram of the size distribution of Pt NPs, (**B**–**D**) EDX elemental mapping images of Pt, Ce, and O, respectively.

**Figure 3:**
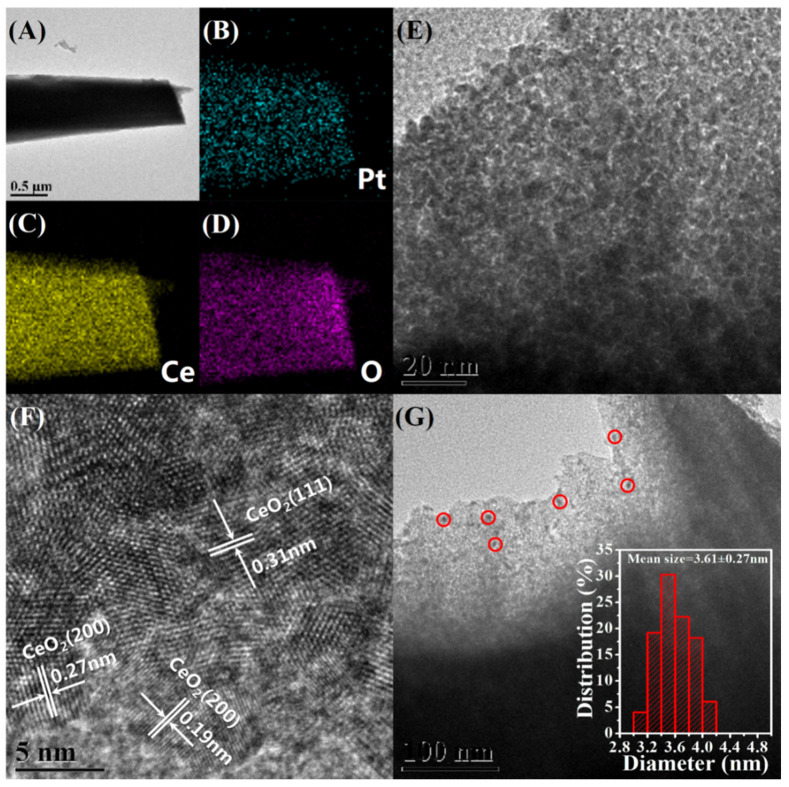
Morphology and structure of Pt(EG)@CeO_2_ (N_2_-Air). (**A**,**E**) TEM images, (**F**) HRTEM images, (**G**) the distribution of Pt NPs and the inset is histogram of the size distribution of Pt NPs, (**B**–**D**) EDX elemental mapping images of Pt, Ce, and O, respectively.

Transmission electron microscopy (TEM) is used to investigate the distribution and size of Pt NPs in the Pt@Ce-BTC as well as the microstructure of Pt@CeO_2_. Monodisperse Pt NPs are successfully immobilized inside the Ce-BTC frameworks without any obvious aggregation based on TEM observations (Figure S4A,B, Supporting Information). As illustrated in the Figure S4C,D (Supporting Information), the mean size of these Pt NPs is 3.05 ± 0.26 nm in Pt(EG)@Ce-BTC and 2.23 ± 0.24 nm in Pt(Et)@Ce-BTC, calculated by counting more than 300 particles for each sample. After pyrolyzing, the resulting Pt@CeO_2_ products inherit the microrod morphology, which is further confirmed by TEM (Figure 2 and Figure 3, and Figures S5 and S6 in the Supporting Information). As for Pt(EG)@CeO_2_ (Air), a rod-like structure with numerous three-dimensional penetrating channels can be observed in Figure 2A. Importantly, Figure 2E reveals that the rod-like structure is composed of many interconnected CeO_2_ nanocrystallites instead of smooth Ce-BTC crystal. The assembly of CeO_2_ nanocrystallites results in the formation of cavities and fissures, also proved by SEM above. The lattice fringes of CeO_2_ nanocrystallites are observed clearly by high-resolution transmission electron microscopy (HRTEM) depicted in Figure 2F. The representative interplanar spacings are around 0.31 and 0.27 nm, matching well with the (111) and (200) planes of ceria phase. As marked by red circles in Figure 2G, it is noteworthy that highly dispersed Pt NPs are well immobilized by the formed CeO_2_ nanocrystallites after calcining at 350 °C under air. According to the inset histogram in Figure 2G, the mean size of Pt NPs is 3.89 ± 0.28 nm, which is slightly bigger than that of Pt(EG)@Ce-BTC (3.05 ± 0.26 nm), indicating that the Pt NPs almost maintain the similar ultrasmall size during the pyrolysis process. Namely, the migration and aggregation of Pt NPs will be prohibited utmostly due to the confinement effect of Ce-BTC framework. Furthermore, with the help of energy dispersive X-ray spectroscopy (EDX) elemental mappings (Figure 2B–D), the Ce, O, and Pt elements are uniformly distributed in the rod-like structure, demonstrating that Pt is highly dispersed throughout the CeO_2_ matrix. Interestingly, compared with Pt(EG)@CeO_2_ (Air), Pt(EG)@CeO_2_ (N_2_-Air) shows some distinctive characteristics. Specially, denser accumulation of CeO_2_ nanocrystallites provides more powerful confinement effect to limit the growth of Pt NPs, and thus the size distribution of Pt NPs in Pt(EG)@CeO_2_ (N_2_-Air) (mean size = 3.61 ± 0.27 nm) is more similar to 3.05 ± 0.26 nm (Pt(EG)@Ce-BTC) than 3.89 ± 0.28 nm (Pt(EG)@CeO_2_ (Air)) (Figure 3E,G). From the HRTEM image in Figure 3F, the CeO_2_ nanocrystallites exposes (220) plane besides (111) and (200). Moreover, Pt(EG)@CeO_2_ (N_2_-Air) holds the integrated shape of microrod and uniform distribution of Pt even after two-step pyrolysis (Figure 3A–D). Additionally, Pt(Et)@CeO_2_ (Air) and Pt(Et)@CeO_2_ (N_2_-Air) possess the same features as Pt(EG)@CeO_2_ (Air) and Pt(EG)@CeO_2_ (N_2_-Air) (Figures S5 and S6, Supporting Information). In conclusion, the special three-dimensional penetrating channels can not only enhance the mass transfer of reactants but also suppress the aggregation of Pt NPs, which is beneficial for VOC diffusion and activation on catalysts [25].

**Figure 4:**
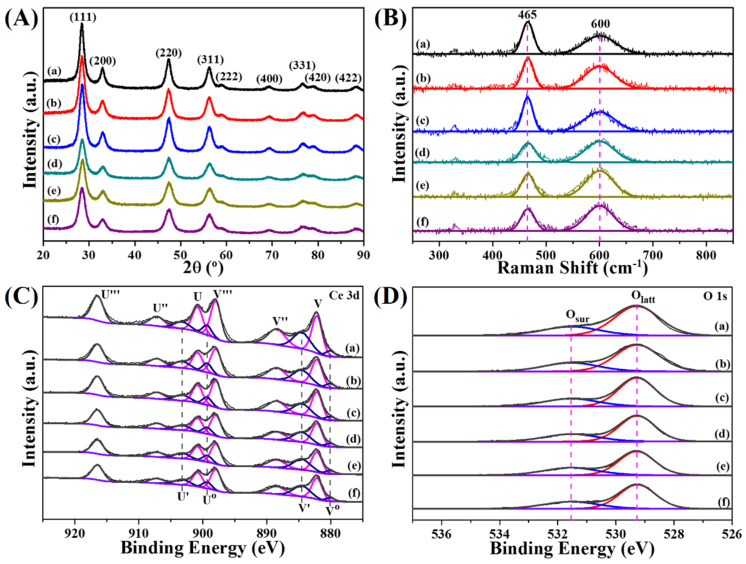
Crystal structure and surface chemical properties of various functional MOF derivatives. (**A**) XRD patterms, (**B**) UV Raman spectra and XPS spectra of (**C**) Ce 3d and (**D**) O 1s for functional MOF derivatives: (a) CeO_2_ (Air), (b) Pt(EG)@CeO_2_ (Air), (c) Pt(Et)@CeO_2_ (Air), (d) CeO_2_ (N_2_-Air), (e) Pt(EG)@CeO_2_ (N_2_-Air), (f) Pt(Et)@CeO_2_ (N_2_-Air).

The XRD patterns of pyrolysis samples are exhibited in Figure 4A. After pyrolyzing, all diffraction peaks are in excellent agreement with the face-centered cubic phase of ceria (JCPDS no. 34-0394) without any signal of Ce-BTC or Pt NPs, suggesting the complete decomposition of MOF precursors and well dispersion of Pt species. Additionally, the XRD peaks appear at 2*θ* = 28.55°, 33.08°, 47.48°, 56.33°, 59.09°, 69.40°, 76.70°, 79.07°, and 88.41°, corresponding to the (111), (200), (220), (311), (222), (400), (331), (420), and (422) planes, respectively. Notably, the two-step pyrolysis samples show a lower average crystallite size than the one-step ones, which are calculated from the half peak width of the ceria (111) plane using Scherrer formulation (Table S2, Supporting Information).

The specific surface areas and pore size distributions of the MOF derivatives are confirmed by N_2_ sorption measurements. Two different kinds of N_2_ adsorption–desorption isotherms are observed from the derivatives depending on the pyrolyzing procedure. As shown in Figure S7A, the derivatives prepared by one-step pyrolysis exhibit typical type ΙV isotherms with H3 hysteresis loops, indicating the irregularly mesoporous structure. However, the isotherms of the two-step pyrolysis samples are identified as type ΙV with H4 hysteresis loops (Figure S7C), revealing the hierarchically mesoporous and microporous structure. As summarized in Table S2 in the Supporting Information, the BET surface areas of the derivatives fall within the range of 123 to 150 m^2^ g^−1^, which is higher than the values reported previously [26,27]. The pore size distributions of the derivatives are calculated by the DFT method. It is found that the one-step pyrolysis samples are all mesoporous with irregular size distribution which is mainly at 2.9 nm and 35 nm (Figure S7B). As for two-step pyrolysis samples, a combination of micropores (mainly at 0.5 nm and 1.3 nm) and mesopores (2.5 nm) has been observed verifying existence of hierarchically meso- and microporous structure (Figure S7D).

### 3.2. Surface Chemical Properties

X-ray photoelectron spectroscopy (XPS) is used to investigate the surface composition and chemical states of the pyrolysis samples. In order to obtain detailed information of surface oxygen species, the XPS spectra of O 1 s are analyzed (Figure 4D). Two main peaks detected at 529.3 eV and 531.5 eV are assigned to lattice oxygen (*O_latt_*) and surface absorbed oxygen (*O_sur_*), respectively [28]. The *O_sur_*/(*O_sur_* + *O_latt_*) ratio is estimated to represent the content of *O_sur_* species and the values are listed in Table S3 in the Supporting Information. The results show that the ratio ranks in the following order: Pt(EG)@CeO_2_ (N_2_-Air) (32.3%) > Pt(Et)@CeO_2_ (N_2_-Air) (30.9%) > CeO_2_ (N_2_-Air) (30.3%) > Pt(EG)@CeO_2_ (Air) (29.2%) > Pt(Et)@CeO_2_ (Air) (28.7%) > CeO_2_ (Air) (27.7%). The two-step pyrolysis samples show a remarkable higher *O_sur_*/(*O_sur_* + *O_latt_*) ratio than the one-step ones, indicating that more *O_sur_* species exists on two-step pyrolysis samples surface.

The Ce 3d spectra of CeO_2_ and Pt@CeO_2_ samples are illustrated in Figure 4C. Herein, two series of labels (V and U) are used to denote the five pairs of spin-orbit components assigned to 3d_5/2_ and 3d_3/2_. The six peaks marked with V, V′′, V′′′, U, U′′, and U′′′ are characteristic of Ce^4+^ while the other four peaks labelled as V^0^, V′, U^0^, and U′ are attributed to Ce^3+^. Generally, the existence of Ce^3+^ is closely associated with the formation of oxygen vacancies on the ceria surface [29]. As shown in Table S3 in the Supporting Information, the fraction of Ce^3+^ in two-step pyrolysis samples (26.0%, 27.9%, and 27.1% for CeO_2_ (N_2_-Air), Pt(EG)@CeO_2_ (N_2_-Air), and Pt(Et)@CeO_2_ (N_2_-Air), respectively) is larger than that in one-step pyrolysis samples (23.3%, 25.7%, and 23.9% for CeO_2_ (Air), Pt(EG)@CeO_2_ (Air), and Pt(Et)@CeO_2_ (Air), respectively), reflecting that more oxygen vacancies are created on the surface of two-step pyrolysis samples.

It is well-established that the surface oxygen vacancy plays a crucial role in activation of gas phase oxygen and migration of active oxygen species [30,31,32]. Consequently, for the purpose of determining the relative concentration of oxygen vacancies accurately, UV Raman technique is employed due to its excellent sensitivity to defect sites in ceria [33,34]. Figure 4B gives the UV Raman spectra of pyrolysis samples. Two strong peaks at 465 and 600 cm^−1^ are ascribed to the symmetric stretching vibrations mode (F_2g_) of the ceria fluorite phase and the defect-induced mode (D) related to oxygen vacancies, respectively [34,35,36]. In general, the ratio of the integral peak areas at 600 and 465 cm^−1^ (A_600_/A_465_) is utilized to represent the relative oxygen vacancies concentration [37]. Compared with the samples merely calcined under air, two-step pyrolysis samples possess more surface oxygen vacancies, which is proved by the higher value of A_600_/A_465_ (Table S3 in the Supporting Information). Combining the results of XPS and UV Raman, it can be concluded that the two-step pyrolysis route helps to increase oxygen vacancies as well as *O_sur_* species, and this enhancement will facilitate the catalytic oxidation reaction [38].

### 3.3. Catalytic Activity, Reusability and Durability

**Figure 5:**
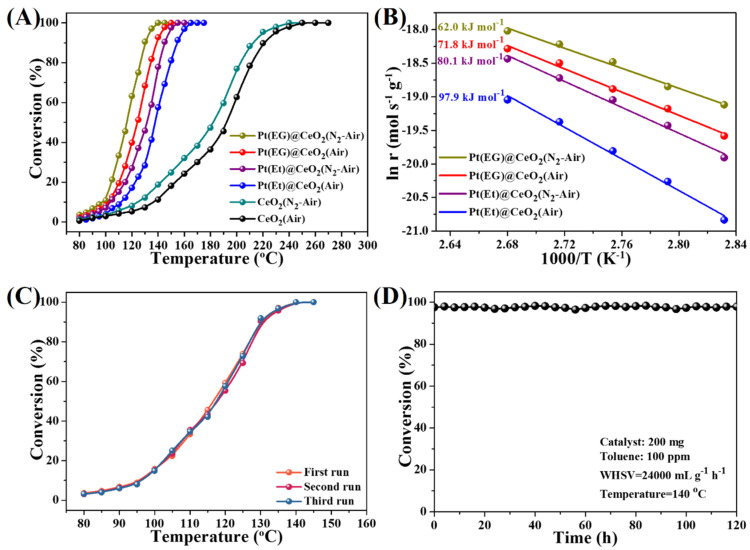
(**A**) Conversion of toluene over various functional MOF derivatives. Catalyst: 200 mg; Toluene concentration: 100 ppm; WHSV: 24,000 mL g^−1^ h^−1^. (**B**) Arrhenius plots for the oxidation of toluene over various functional MOF derivatives. (**C**) Conversion of toluene over Pt(EG)@CeO_2_ (N_2_-Air) with three consecutive cycles. (**D**) Long-term durability test over Pt(EG)@CeO_2_ (N_2_-Air).

The catalytic activities of MOF derivatives prepared by two different pyrolysis routes are evaluated for toluene oxidation which is operated in a temperature range of 80–280 °C (Figure 5A). For comparison, the temperatures of *T_10_*, *T_50_* and *T_90_* (corresponding to the toluene conversion of 10%, 50%, and 90%, respectively) are summarized in Table S4 in the Supporting Information. From Figure 5A and Table S4, it is worth pointing out that the two-step pyrolysis samples display better activities than the homologous one-step pyrolysis samples. Particularly, among the four Pt@CeO_2_ catalysts, Pt(EG)@CeO_2_ (N_2_-Air) shows the highest catalytic activity with *T_90_* at 130 °C, lower than that of Pt(EG)@CeO_2_ (Air) (138 °C), Pt(Et)@CeO_2_ (N_2_-Air) (145 °C), or Pt(Et)@CeO_2_ (Air) (154 °C). Furthermore, the Arrhenius plots (Figure 5B) for toluene oxidation at low conversion (less than 10%) are used to calculate the apparent activation energies (*E_a_*), and the results are listed in Table S4 in the Supporting Information. The *E_a_* for toluene oxidation over the four Pt@CeO_2_ catalysts increase in the following order: Pt(EG)@CeO_2_ (N_2_-Air) (60.2 kJ mol^−1^) < Pt(EG)@CeO_2_ (Air) (71.8 kJ mol^−1^) < Pt(Et)@CeO_2_ (N_2_-Air) (80.1 kJ mol^−1^) < Pt(Et)@CeO_2_ (Air) (97.9 kJ mol^−1^). The two-step pyrolysis samples show lower *E_a_* than the homologous one-step pyrolysis samples, indicating that the complete oxidation of toluene is easier over the two-step pyrolysis samples, which is consistent with the comparison of the catalytic activities. In order to explore the reusability of Pt@CeO_2_ derived from Pt@Ce-BTC, the three consecutive cycles from 80 to 180 °C are carried out and the results are shown in Figure 5C and Figure S8 (Supporting Information). The toluene conversion curves for the three consecutive runs are almost overlapping with each other over all of the Pt@CeO_2_ samples, demonstrating that these Pt@CeO_2_ catalysts are reusable after reacting for three runs. Additionally, the long-term durability test over the most promising catalyst Pt(EG)@CeO_2_ (N_2_-Air) is evaluated. As shown in Figure 5D, the toluene conversion successfully maintains at 97% during 120 h reaction process (reaction conditions: 200 mg catalyst, 100 ppm toluene, temperature at 140 °C), exhibiting excellent durability. These reusability and durability test results reveal that the Pt@CeO_2_ composites prepared by one-step or two-step pyrolysis display stable activities for toluene oxidation.

### 3.4. Effect of Oxygen Activation Ability on Toluene Oxidation

On the basis of the well-established Mars–van Krevelen mechanism, the toluene oxidation pathway over Pt@CeO_2_ probably basically includes three steps [39,40,41,42]: (1) the adsorption of toluene molecule; (2) the adsorption and activation of gas phase oxygen; (3) the reaction of adsorbed toluene molecule and active oxygen. It has been widely accepted that the gaseous oxygen activation is a necessary step [43,44]. Therefore, the oxygen activation ability of the Pt@CeO_2_ catalysts has a significant influence on the catalytic activity. To explore the ability of adsorption and activation of gas phase oxygen over the obtained Pt@CeO_2_ catalysts, in situ UV Raman is conducted under N_2_ (Figure S9A,C,E,G in the Supporting Information) or dry air (Figure S9B,D,F,H in the Supporting Information). The original peaks at 465 cm^−1^ and 600 cm^−1^ corresponding to F_2g_ band and oxygen vacancies, respectively, keep the intense signals during the in-situ UV Raman test. The values of A_600_/A_465_ are also utilized to represent the relative concentration of oxygen vacancies under different temperature and atmosphere. Interestingly, similar changing trends of A_600_/A_465_ are observed in various atmospheres over the Pt@CeO_2_ samples. As shown in Table S5 (Supporting Information), for all of Pt@CeO_2_, the A_600_/A_465_ values decline rapidly with the temperature rising from 30 °C to 120 °C and keep stable at temperatures above 130 °C. However, the decrease of oxygen vacancies concentration is caused by various effects under different atmospheres. In N_2_ atmosphere, the annihilation of oxygen vacancies is due to thermal expansion and mode softening [45]. While in dry air, besides the thermal expansion effect, the oxygen vacancies also adsorb gaseous oxygen molecules and transform them into active oxygen species, such as superoxide and peroxide species [31,34]. These active oxygen species occupy the oxygen vacancies and accumulate on the catalyst surface, leading to the disappearance of oxygen vacancies. Briefly speaking, the decreases of A_600_/A_465_ values under dry air are attributed to the combination of thermal effect and gaseous oxygen activation.

**Figure 6:**
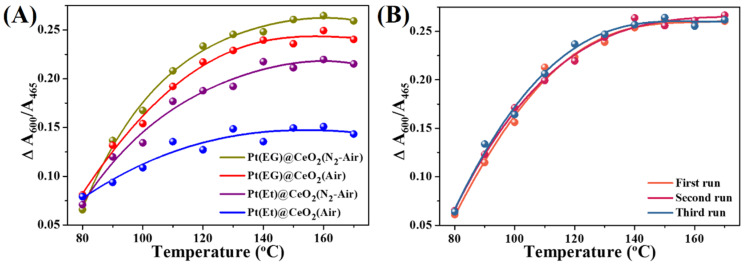
(**A**) Gaseous oxygen activation ability of various functional MOF derivatives. (**B**) Gaseous oxygen activation ability of Pt(EG)@CeO_2_ (N_2_-Air) in the three runs.

Herein, the A_600_/A_465_ value at 30 °C is set as original concentration of oxygen vacancies, denoted as *C_X, 30 °C_* (*X* = N_2_ or air, respectively). The decrement (ΔA_600_/A_465_) at different temperature and atmosphere conditions is calculated by (*C_X, 30 °C_ − C_X, Y_*) (*Y* represents temperature from 80 °C to 170 °C). For example, the decrement at 80 °C under N_2_ is calculated by (*C_N2, 30 °C_ − C_N2, 80 °C_*). Figure S10 (Supporting Information) shows the ΔA_600_/A_465_ value at different temperature and atmosphere. Hence, the thermal effect is quantified by the decrement under N_2_ which is marked as Δ_N2_ A_600_/A_465_, corresponding to the cyan portions in Figure S10 (Supporting Information). While Δ_Air_ A_600_/A_465_ reveals the quantity of thermal effect plus oxygen activation, corresponding to the violet portions in Figure S10 (Supporting Information). Specially, the Δ_N2_ A_600_/A_465_ is subtracted from Δ_Air_ A_600_/A_465_ to eliminate the thermal effect, and the (Δ_Air_ A_600_/A_465_ − Δ_N2_ A_600_/A_465_) value reflects the oxygen activation ability of the Pt@CeO_2_ catalysts, corresponding to the magenta grid in Figure S10 (Supporting Information). The relationship between (Δ_Air_ A_600_/A_465_ − Δ_N2_ A_600_/A_465_) value and temperature of different Pt@CeO_2_ samples is illustrated in Figure 6A. It is noteworthy that Pt(EG)@CeO_2_ (N_2_-Air) possesses the highest (Δ_Air_ A_600_/A_465_ − Δ_N2_ A_600_/A_465_) value, indicating the strongest ability to adsorb and activate gaseous oxygen. Benefitting from the strongest ability of oxygen activation, Pt(EG)@CeO_2_ (N_2_-Air) can supply the most active oxygen species for toluene oxidation, thus exhibits the best catalytic activities. Moreover, the values of (Δ_Air_ A_600_/A_465_ − Δ_N2_ A_600_/A_465_) decrease in the following order: Pt(EG)@CeO_2_ (N_2_-Air) > Pt(EG)@CeO_2_ (Air) > Pt(Et)@CeO_2_ (N_2_-Air) > Pt(Et)@CeO_2_ (Air), which is well consistent with catalytic activities. Additionally, the three recycle Raman tests over the best-performing catalyst Pt(EG)@CeO_2_ (N_2_-Air) are carried out under N_2_ or air, respectively, in order to depict its excellent reusability for toluene oxidation. Figure S11 (Supporting Information) displays the original in situ UV Raman spectra for the three recycle runs, and the oxygen activation ability of Pt(EG)@CeO_2_ (N_2_-Air) in the three runs (Figure 6B) is obtained by the above method. As shown by Figure 6B, there is no significant decline of (Δ_Air_ A_600_/A_465_ − Δ_N2_ A_600_/A_465_) value within three recycle runs, suggesting that the Pt(EG)@CeO_2_ (N_2_-Air) maintains its outstanding ability of adsorption and activation gaseous oxygen. Therefore, the steady supply of active oxygen species guarantee the excellent reusability of Pt(EG)@CeO_2_ (N_2_-Air).

## 4. Conclusions

In summary, the functional ceria-based MOF derivatives have been synthesized by the one-step or two-step in situ pyrolysis of ceria-based MOF precursors. The functional MOF derivatives still maintain the morphology of MOF precursors, and the Pt NPs keep away from the migration and aggregation as well as uniformly distribute throughout the CeO_2_ matrix after in situ pyrolysis process. Compared with the one-step pyrolysis strategy, the functional MOF derivatives prepared via two-step pyrolysis exhibit novel hierarchical pore structure and possess higher concentration of surface oxygen vacancy, thereby significantly enhancing mass transfer and activation properties. More importantly, the gaseous oxygen activation ability of the MOF derivatives has been promoted by two-step pyrolysis strategy, which plays an important role in toluene oxidation. The two-step pyrolysis composites perform higher activity in the toluene oxidation reaction than one-step pyrolysis samples, and Pt(EG)@CeO_2_ (N_2_-Air) displays the best catalytic performance (*T_50_* = 116 °C, *T_90_* = 130 °C). Furthermore, the functional MOF derivatives are perfectly stable during toluene oxidation, as demonstrated by the reusability and long-term durability test. On the one hand, this work provides different in situ pyrolysis strategies to prepare functional MOF derivatives with unique structure, which can further expand the application of MOFs. On the other hand, we offer fundamental insights to quantify the ability of adsorption and activation of gas phase oxygen over ceria-based catalysts.

## Supplementary Materials

The following are available online at https://www.mdpi.com/2079-4991/10/5/983/s1, Figure S1: XRD patterns of Ce-BTC and Pt@Ce-BTC., Figure S2: Nitrogen adsorption-desorption isotherms and pore-size distributions of Ce-BTC and Pt@Ce-BTC, Figure S3:TGA curve of Ce-BTC, Figure S4: TEM images and Pt NPs size distribution histogram of Pt@Ce-BTC, Figure S5: Morphology and structure of Pt(Et)@CeO_2_ (Air), Figure S6: Morphology and structure of Pt(Et)@CeO_2_ (N_2_-Air), Figure S7: Nitrogen adsorption-desorption isotherms and pore-size distribution of the CeO_2_ and Pt@CeO_2_, Figure S8: Conversion of toluene over Pt@CeO_2_ with three consecutive cycles, Figure S9: In situ UV Raman spectra, Figure S10: The oxygen activation ability over Pt@CeO_2_, Figure S11: In situ UV Raman of Pt(EG)@CeO_2_ (N_2_-Air), Table S1: Surface area and pore volume data for Ce-BTC and Pt@Ce-BTC, Table S2: Structural properties of various functional MOFs derivatives, Table S3: Surface chemical properties of various functional MOFs derivatives, Table S4: Catalytic activities and apparent activation energies for various functional MOFs derivatives, Table S5:The relative concentration of oxygen vacancies data.
