# Supplementary material for: CeO2-Supported Pt Catalysts Derived from MOFs by Two Pyrolysis Strategies to Improve the Oxygen Activation Ability
Source: Nanomaterials (Basel). 2020 May 21;10(5):983. doi: 10.3390/nano10050983 (PMC7279553; doi:10.3390/nano10050983)
Supplement: Supplementary file 1 [file nanomaterials-10-00983-s001.pdf]

nanomaterials

Supplementary Information

CeO_2-Supported Pt Catalysts Derived from MOFs by Two Pyrolysis Strategies to Improve the Oxygen Activation Ability

Xueqing Zhu [1], Hui He [1], Yanxia Li [1], Haoyuan Wu [1], Mingli Fu [1,2,3,*], Daiqi Ye [1,2,3], Junliang Wu [1,2], Haomin Huang [1], Yun Hu [1], and Xiaojun Niu [1]

1 School of Environment and Energy, South China University of Technology, Guangzhou 510006, China; 201720141893@mail.scut.edu.cn (X.Z.); eshehui@mail.scut.edu.cn (H.H.); 201821035341@mail.scut.edu.cn (Y.L.); 201630793250@mail.scut.edu.cn (H.W.); cedqye@scut.edu.cn (D.Y.); ppjl@scut.edu.cn (J.W.); huanghm@scut.edu.cn (H.H.); huyun@scut.edu.cn (Y.H.); xjniu@scut.edu.cn (X.N.)

2 Guangdong Provincial Key Laboratory of Atmospheric Environment and Pollution Control, Guangzhou 510006, China

3 National Engineering Laboratory for VOCs Pollution Control Technology and Equipment, South China University of Technology, Guangzhou 510006, China

* Correspondence: mlfu@scut.edu.cn; Tel.: +86-20-39380508

Received:21 April 2020; Accepted: 16 May 2020; Published: 21 May 2020

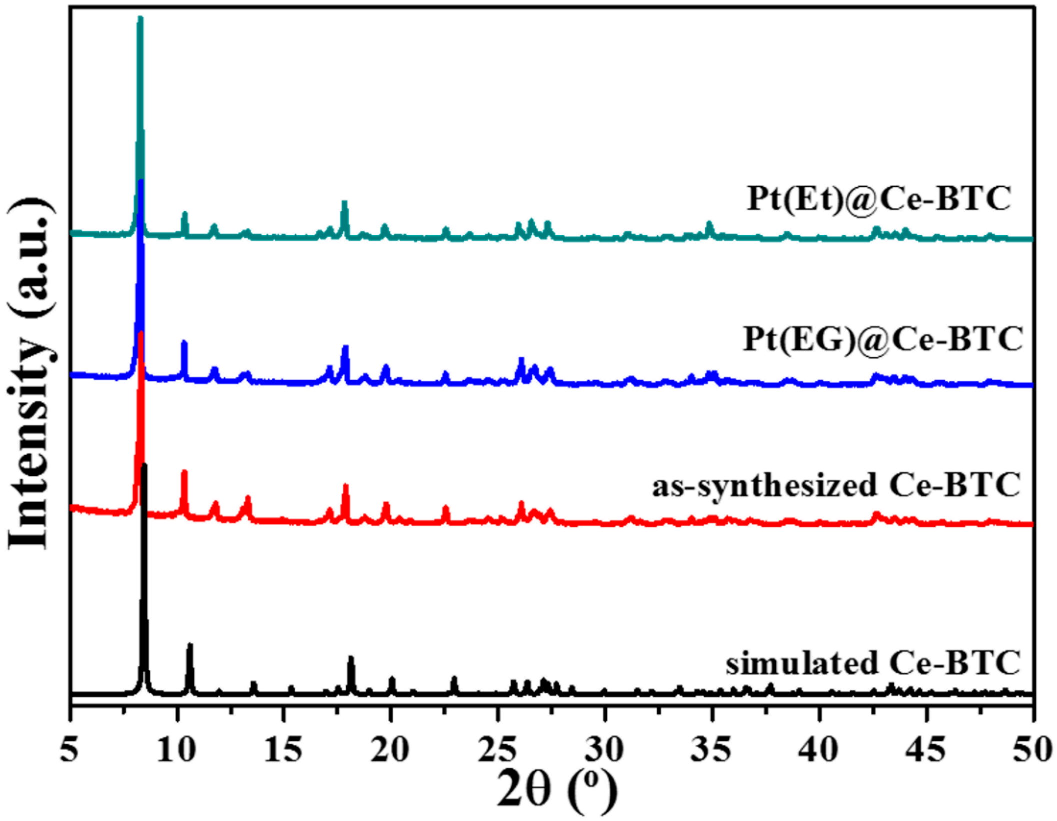

Figure S1. XRD patterns of as-synthesized Ce-BTC (red), Pt(EG)@Ce-BTC (blue), Pt(Et)@Ce-BTC (dark cyan) and simulated pattern of Ce-BTC (black). The XRD patterns of as-synthesized samples match well with the simulated pattern confirming good crystallinity and purity.

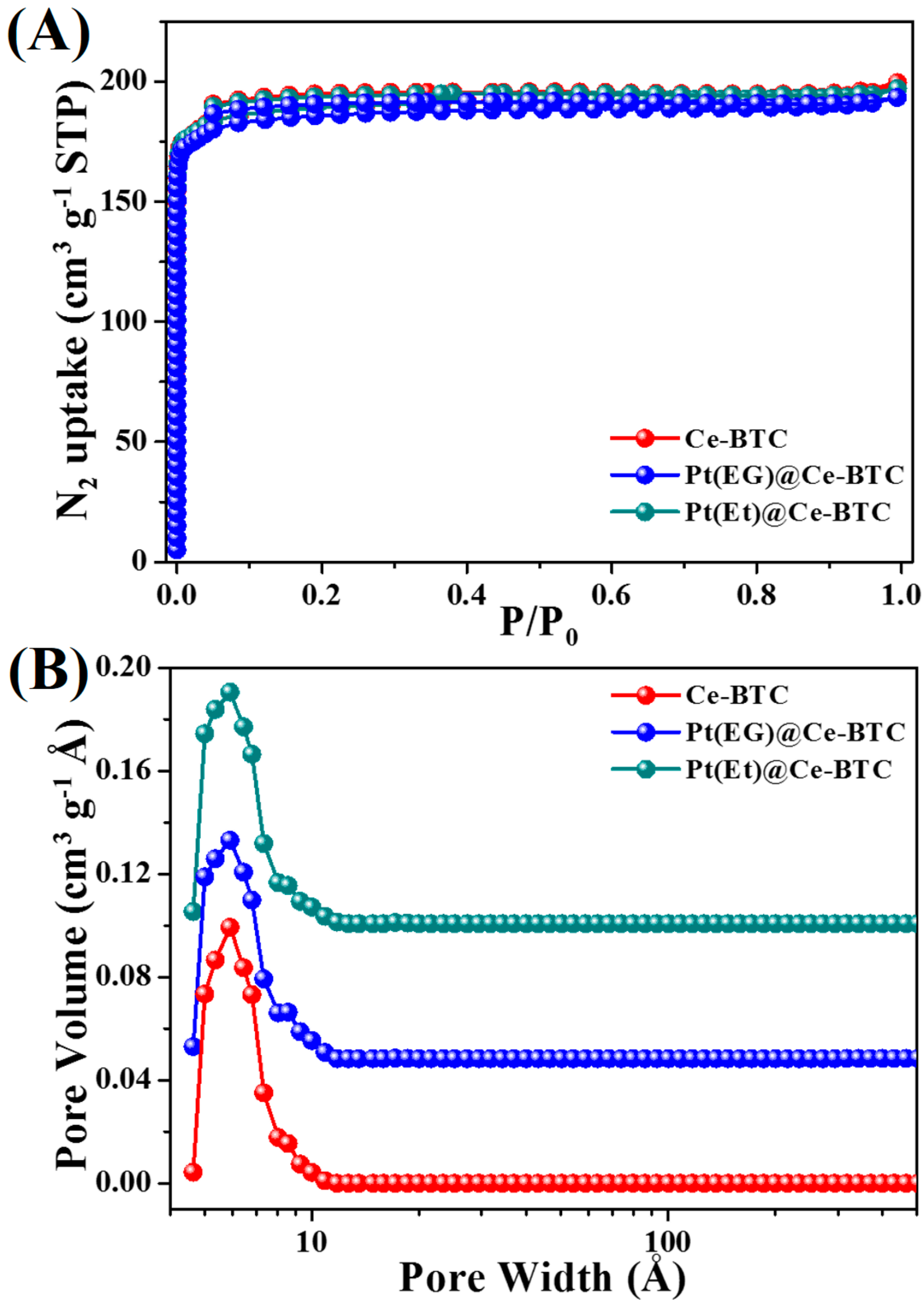

Figure S2. (**A**) Nitrogen adsorption-desorption isotherms for Ce-BTC (red), Pt(EG)@Ce-BTC (blue), Pt(Et)@Ce-BTC (dark cyan) at 77 K. All the as-synthesized samples exhibit classical type I isotherms without hysteresis, suggesting the microporous structures. (**B**) Corresponding pore-size distributions calculated by DFT method.

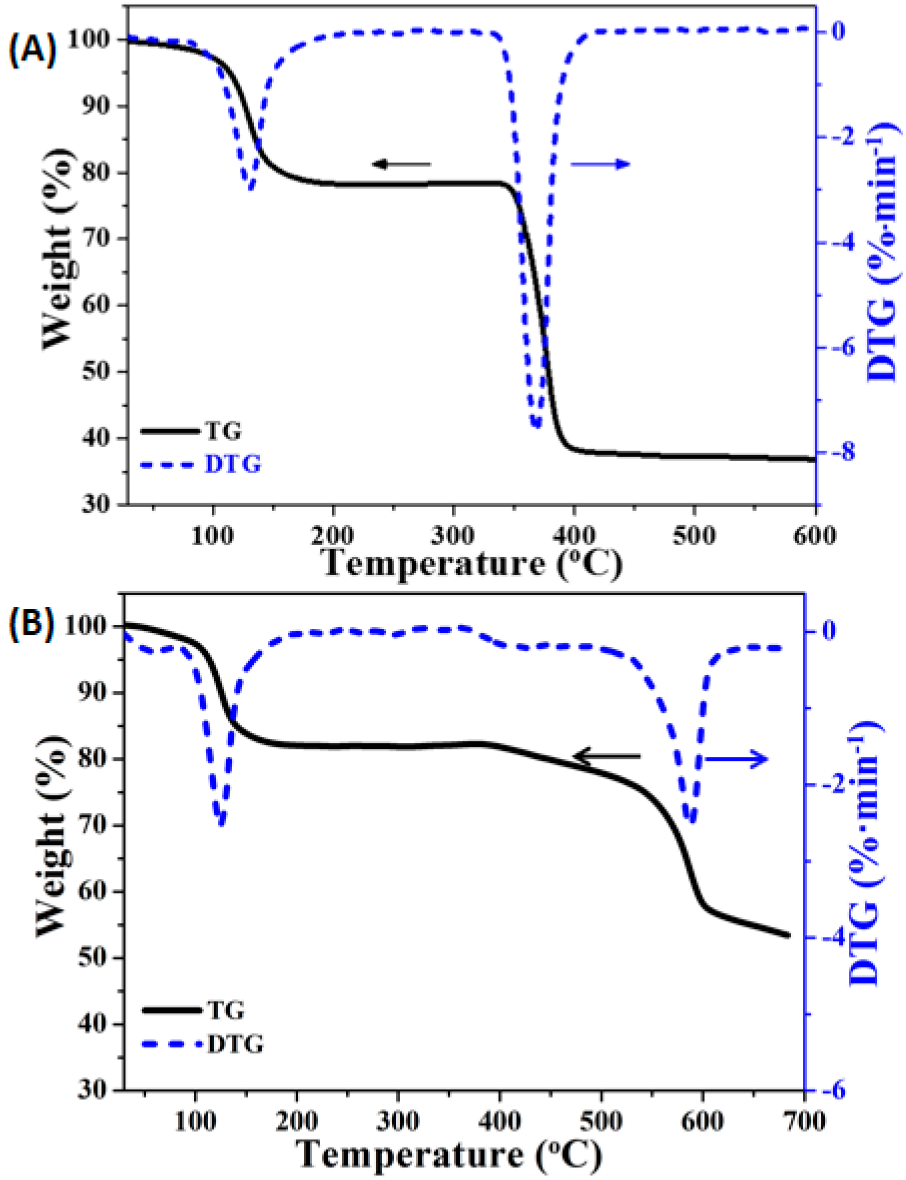

Figure S3. (**A**) TGA curve of as-synthesized pristine Ce-BTC in the 30–600 °C range obtained under dry air. The first weight loss in the 90–170 °C corresponds to the desolvation process. The second weight loss in the 350–400 °C corresponds to the collapse of Ce-BTC framework. (**B**) TGA curve of as-synthesized pristine Ce-BTC in the 30–700 °C range obtained under N_2. The Ce-BTC framework decomposes slowly from 500 °C.

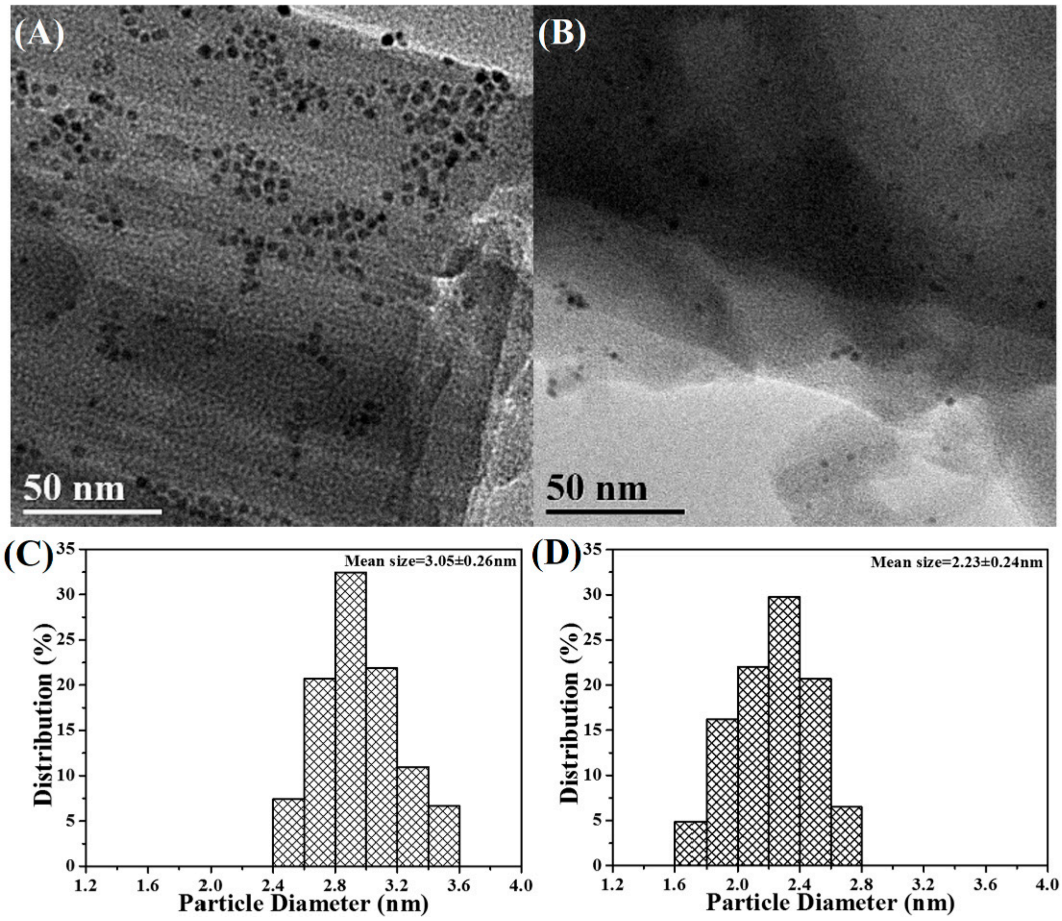

Figure S4. TEM images of (**A**) Pt(EG)@Ce-BTC and (**B**) Pt(Et)@Ce-BTC. Histogram of the size distribution of Pt NPs of (**C**) Pt(EG)@Ce-BTC sample and (**D**) Pt(Et)@Ce-BTC sample.

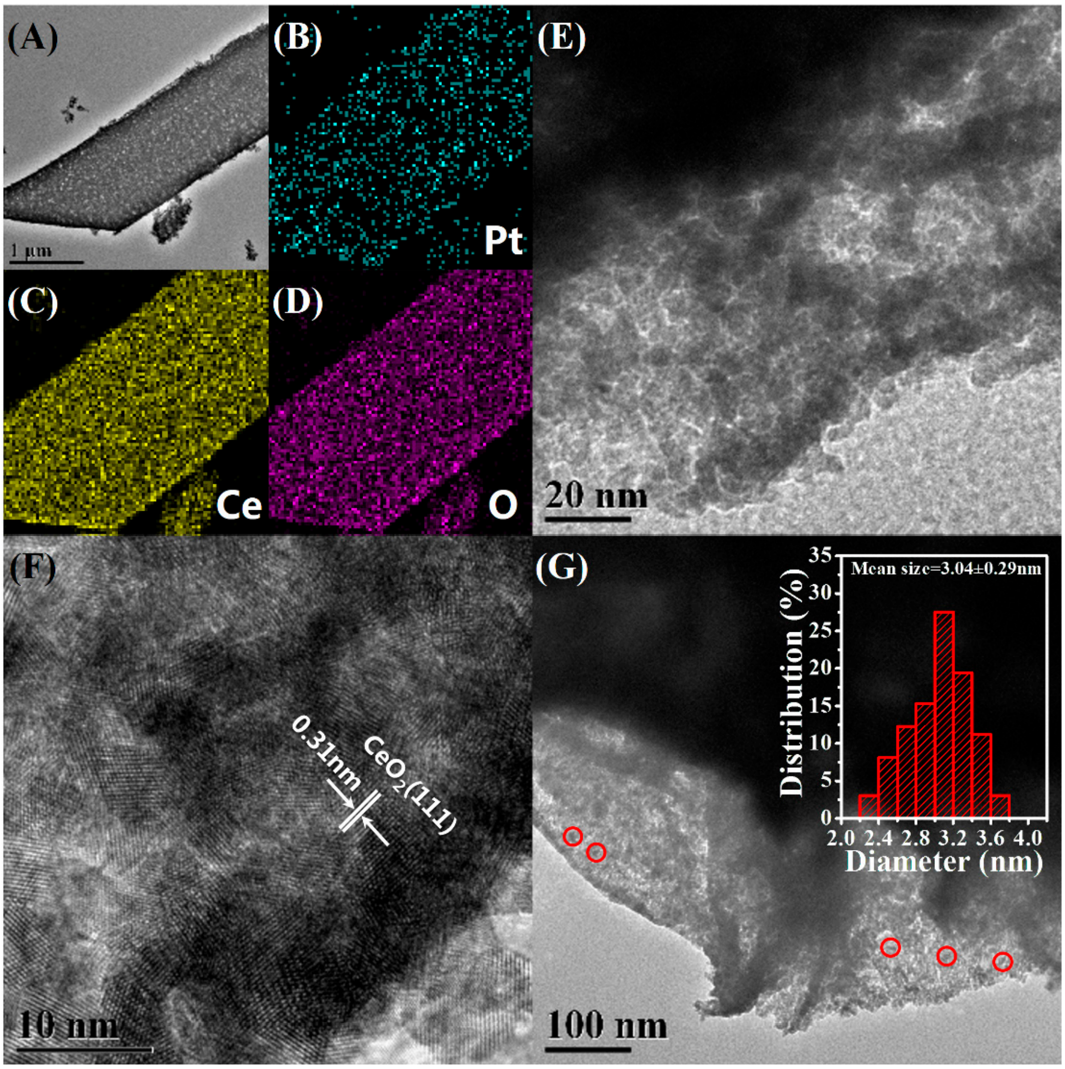

Figure S5. Morphology and structure of Pt(Et)@CeO_2 (Air). (**A**,**E**) TEM images, (**F**) HRTEM images, (**G**) the distribution of Pt NPs and the inset is histogram of the size distribution of Pt NPs, (**B**–**D**) EDX elemental mapping images of Pt, Ce and O, respectively.

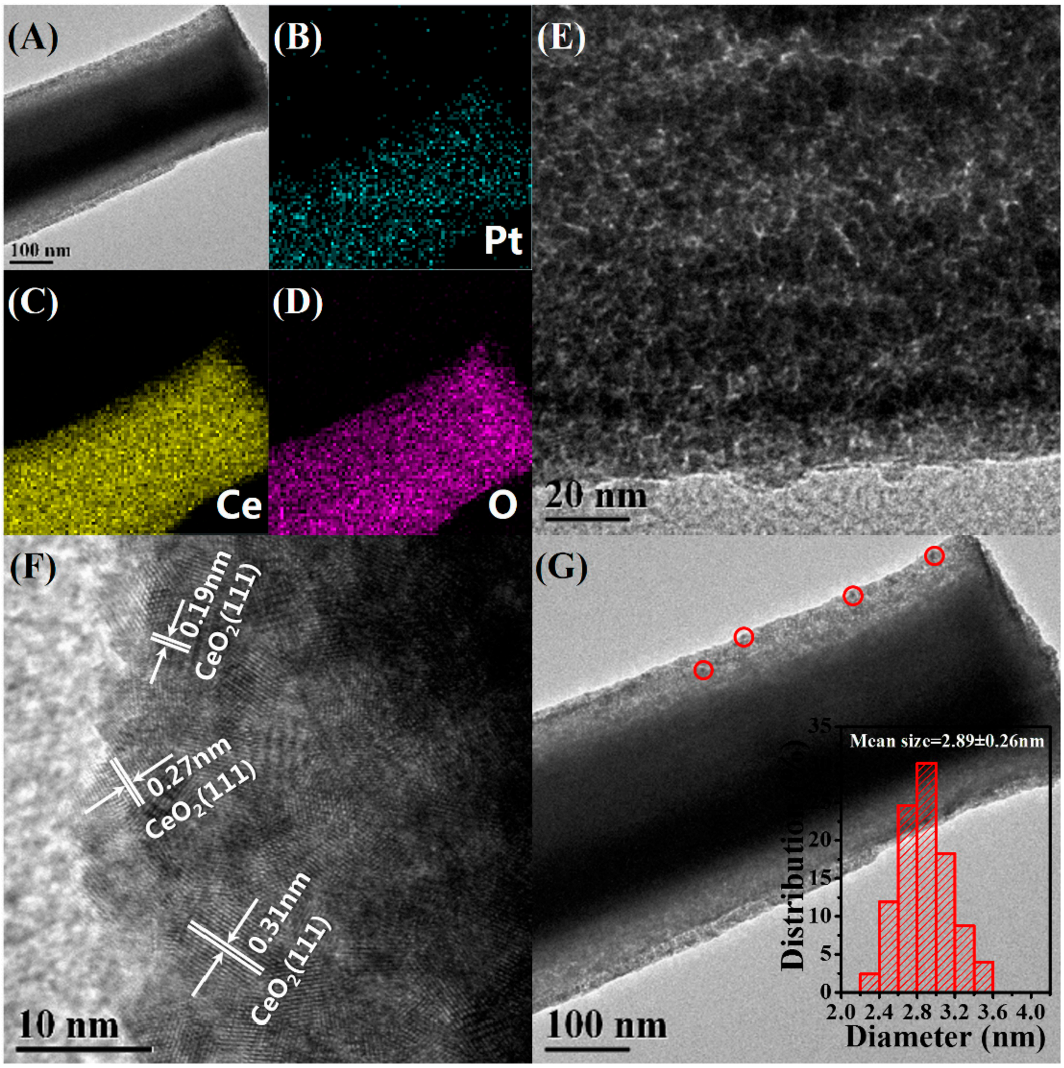

Figure S6. Morphology and structure of Pt(Et)@CeO_2 (N_2-Air). (**A**,**E**) TEM images, (**F**) HRTEM images, (**G**) the distribution of Pt NPs and the inset is histogram of the size distribution of Pt NPs, (**B–D**) EDX elemental mapping images of Pt, Ce and O, respectively.

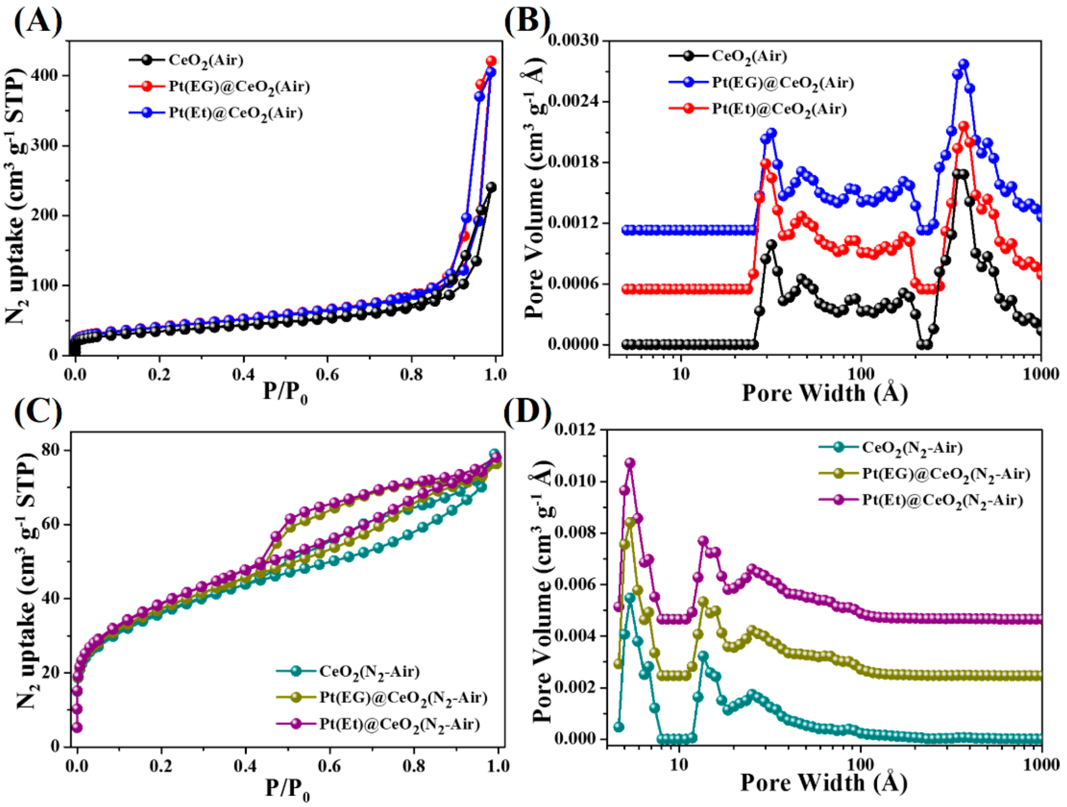

Figure S7. (**A**) Nitrogen adsorption-desorption isotherms for CeO_2 (Air), Pt(EG)@CeO_2 (Air) and Pt(Et)@CeO_2 (Air) at 77 K. All samples exhibit classical type IV isotherms with H3 hysteresis loops, suggesting the mesoporous structures. (**B**) Corresponding pore-size distributions of CeO_2 (Air), Pt(EG)@CeO_2 (Air) and Pt(Et)@CeO_2 (Air) calculated by DFT method. (**C**) Nitrogen adsorption-desorption isotherms for CeO_2 (N_2-Air), Pt(EG)@CeO_2 (N_2-Air) and Pt(Et)@CeO_2 (N_2-Air) at 77 K. All samples exhibit classical type IV isotherms with H4 hysteresis loops, suggesting the hierarchically meso- and microporous structures. (**D**) Corresponding pore-size distributions of CeO_2 (N_2-Air), Pt(EG)@CeO_2 (N_2-Air) and Pt(Et)@CeO_2 (N_2-Air) calculated by DFT method.

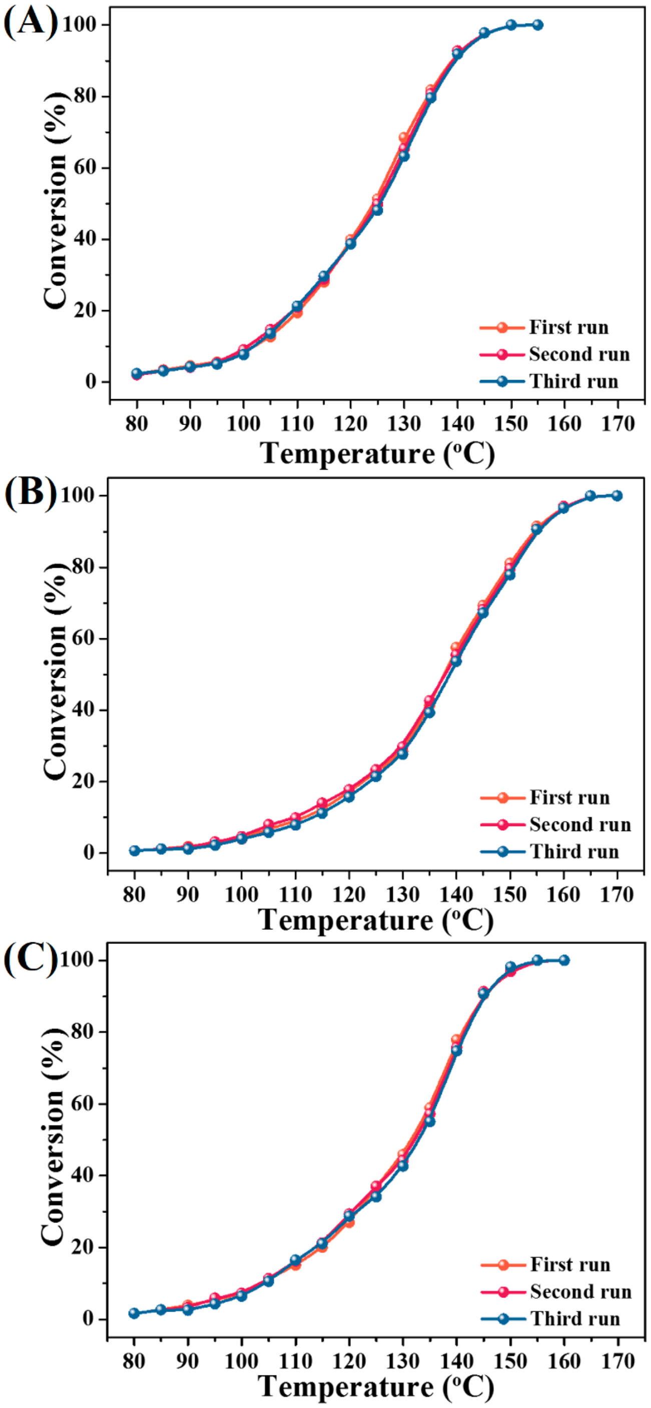

Figure S8. Conversion of toluene over (**A**) Pt(EG)@CeO_2 (Air), (**B**) Pt(Et)@CeO_2 (Air) and (**C**) Pt(Et)@CeO_2 (N_2-Air) with three consecutive cycles. Reaction conditions: 200 mg catalysts + 800 mg quartz sand, 100 ppm toluene, 20% O_2/N_2, temperature from 80 °C to 180 °C. Demonstrating that these Pt@CeO_2 derived from Pt@Ce-BTC are perfect reusability for toluene oxidation.

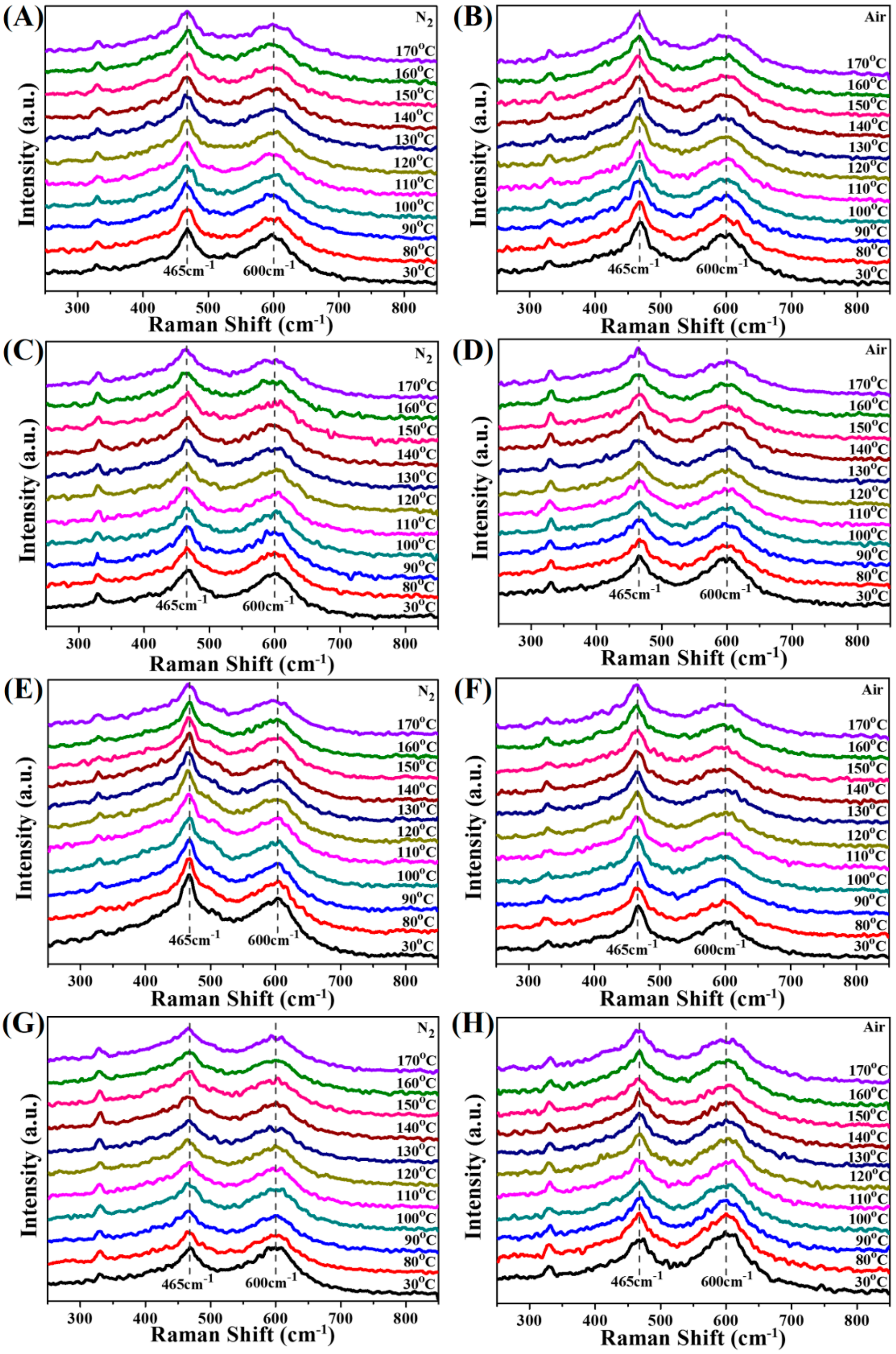

Figure S9. In situ UV Raman under nitrogen (left) and dry air (right). (**A**,**B**) Pt(EG)@CeO_2 (Air), (**C**,**D**) Pt(EG)@CeO_2 (N_2-Air), (**E**,**F**) Pt(Et)@CeO_2 (Air) and (**G**,**H**) Pt(Et)@CeO_2 (N_2-Air). Experimental condition: flow rate = 100 mL min^{-1}, temperature from 80 °C to 170 °C with a ramp rate of 1 °C min^{-1}. The spectrum was recorded by two times 180 s at every 10 °C.

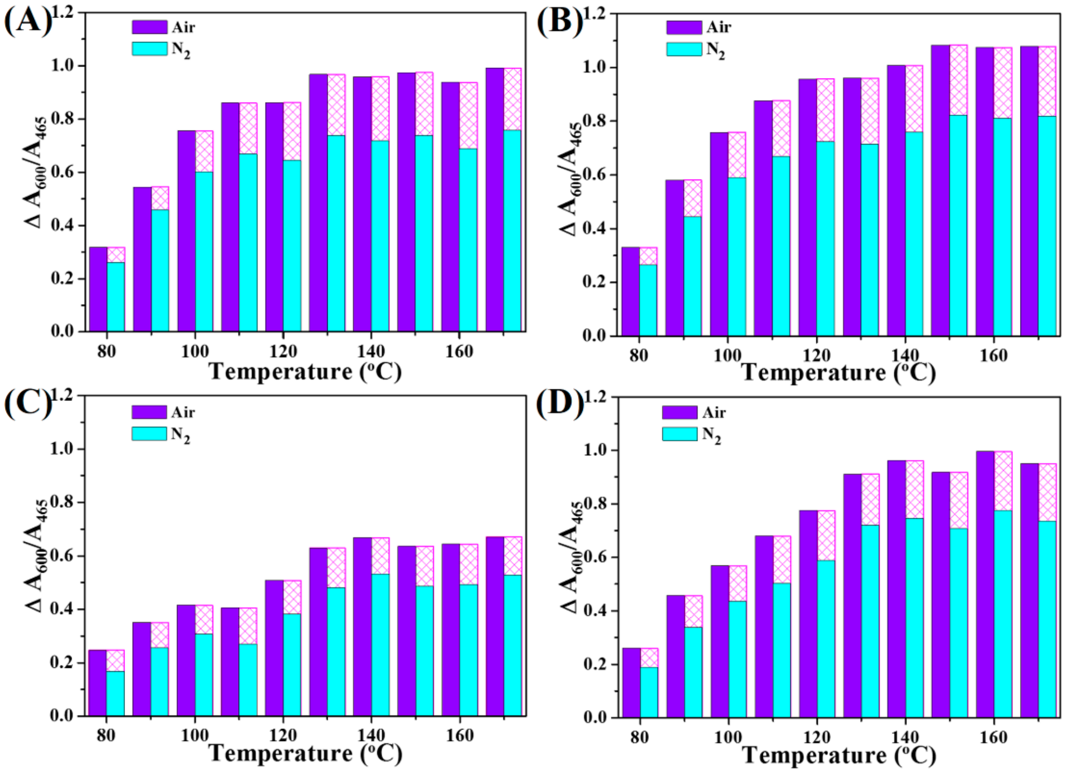

Figure S10. The decrement of A_{600}/A_{465} value at different temperature and atmosphere over (**A**) Pt(EG)@CeO_2 (Air), (**B**) Pt(EG)@CeO_2 (N_2-Air), (**C**) Pt(Et)@CeO_2 (Air) and (**D**) Pt(Et)@CeO_2 (N_2-Air). The magenta grid represented the oxygen activation ability.

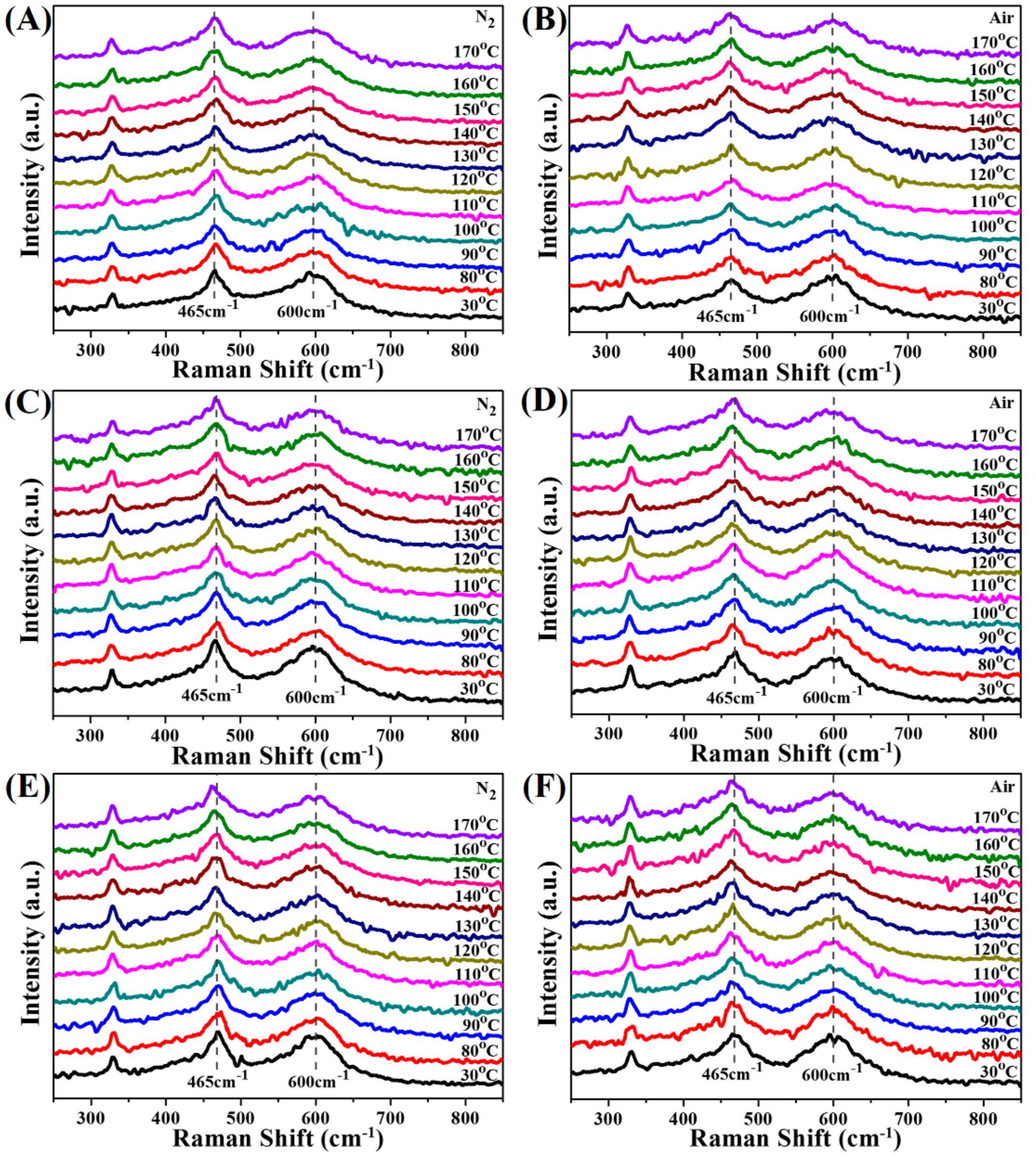

Figure S11. In situ UV Raman spectra of Pt(EG)@CeO_2 (N_2-Air) under nitrogen (left) and dry air (right). (**A**,**B**) the first run, (**C**,**D**) the second run and (**E**,**F**) the third run. Experimental condition: flow rate = 100 mL min^{-1}, temperature from 80 °C to 170 °C with a ramp rate of 1 °C min^{-1}. The spectrum was recorded by two times 180 s at every 10 °C.

Table S1. Surface area and pore volume data for Ce-BTC, Pt(EG)@Ce-BTC and Pt(Et)@Ce-BTC.

Sample	BET SA[a)] [m^2 g^{-1}]	Pore volume[b)] [cm^3 g^{-1}]
Ce-BTC	762.66 ± 0.66	0.309
Pt(EG)@Ce-BTC	745.45 ± 0.21	0.299
Pt(Et)@Ce-BTC	758.63 ± 0.67	0.305

[a)] BET SA is the Brunauer-Emmett-Teller surface area determined from N_2 isotherm; [b)] The pore volume is the total specific pore volume determined by using the adsorption branch of the N_2 isotherm at $P/P_0 = 0.99$.

Table S2. Structural properties of various functional MOFs derivatives.

Sample	Pt content[a)] [%]	Pt NPs mean size[b)] [nm]	BET SA[c)] [m^2 g^{-1}]	Crystallite size[d)] [nm]
CeO_2 (Air)	-	-	123.97	10.4
Pt(EG)@CeO_2 (Air)	0.47	3.89 ± 0.28	147.87	9.3
Pt(Et)@CeO_2 (Air)	0.43	3.04 ± 0.29	145.32	9.5
CeO_2 (N_2-Air)	-	-	131.58	9.1
Pt(EG)@CeO_2 (N_2-Air)	0.44	3.61 ± 0.27	150.35	8.6
Pt(Et)@CeO_2 (N_2-Air)	0.46	2.89 ± 0.26	145.05	8.9

[a)] Pt content is determined by ICP-MS; [b)] Pt NPs mean size is determined by counting more than 300 Pt particles from TEM; [c)] BET SA is the Brunauer-Emmett-Teller surface area determined from N_2 isotherm; [d)] Crystallite size is estimated by the Scherrer equation.

Table S3. Surface chemical properties of various functional MOFs derivatives.

Sample	$O_{sur}/(O_{sur} + O_{latt})$ [%]	Ce^{3+} /(Ce^{3+} + Ce^{4+}) [%]	A_{600}/A_{465}[a)]
CeO_2 (Air)	27.74	23.34	1.54
Pt(EG)@CeO_2 (Air)	29.17	25.66	1.87
Pt(Et)@CeO_2 (Air)	28.69	23.86	1.68
CeO_2 (N_2-Air)	30.33	25.98	2.14
Pt(EG)@CeO_2 (N_2-Air)	32.25	27.92	2.36
Pt(Et)@CeO_2 (N_2-Air)	30.86	27.03	2.25

[a)] A_{600}/A_{465} is the ratio of the integral UV Raman peak areas at 600 and 465 cm^{-1}.

Table S4. Catalytic activities and apparent activation energies for various functional MOFs derivatives.

Sample	T_{10}[a)] [°C]	T_{50}[b)] [°C]	T_{90}[c)] [°C]	Apparent activation energy [kJ mol^{-1}]
CeO_2 (Air)	136	192	220	-
Pt(EG)@CeO_2 (Air)	102	124	138	71.8
Pt(Et)@CeO_2 (Air)	111	137	154	97.9
CeO_2 (N_2-Air)	124	182	212	-
Pt(EG)@CeO_2 (N_2-Air)	97	116	130	62.0
Pt(Et)@CeO_2 (N_2-Air)	103	132	145	80.1

[a)–c)] T_{10}, T_{50} and T_{90} mean the reaction temperature corresponding to toluene conversion of 10%, 50% and 90%, respectively.

Table S5. The relative concentration of oxygen vacancies under different temperature and atmosphere.

Sample	Temperature [°C]	In situ UV Raman atmosphere	
		N_2	Dry air
Pt(EG)@CeO_2 (Air)	30	1.858	1.843
	80	1.599	1.503
	90	1.401	1.254
	100	1.257	1.088
	110	1.191	0.984
	120	1.214	0.982
	130	1.120	0.876
	140	1.139	0.884
	150	1.119	0.869
	160	1.170	0.906
	170	1.100	0.845
Pt(Et)@CeO_2 (Air)	30	1.684	1.670
	80	1.517	1.424
	90	1.427	1.320
	100	1.377	1.255
	110	1.415	1.265
	120	1.303	1.162
	130	1.204	1.041
	140	1.151	1.002
	150	1.198	1.035
	160	1.191	1.026
	170	1.156	0.999
Pt(EG)@CeO_2 (N_2-Air)	30	2.341	2.332
	80	2.077	2.002
	90	1.898	1.752
	100	1.751	1.575
	110	1.674	1.456
	120	1.618	1.375
	130	1.627	1.373
	140	1.582	1.325
	150	1.518	1.249
	160	1.532	1.258
	170	1.522	1.255
Pt(Et)@CeO_2 (N_2-Air)	30	2.267	2.292
	80	2.079	2.033
	90	1.931	1.835
	100	1.833	1.723
	110	1.765	1.612
	120	1.680	1.517
	130	1.548	1.380
	140	1.522	1.329
	150	1.561	1.374
	160	1.492	1.297
	170	1.532	1.341

© 2020 by the authors. Submitted for possible open access publication under the terms and conditions of the Creative Commons Attribution (CC BY) license (http://creativecommons.org/licenses/by/4.0/).
